# Optimization of Site Selection for Emergency Medical Facilities considering the SEIR Model

**DOI:** 10.1155/2022/1912272

**Published:** 2022-04-20

**Authors:** Jingkuang Liu, Liying Cao, Dongyu Zhang, Zibo Chen, Xiaotong Lian, Ying Li, Yingyi Zhang

**Affiliations:** ^1^School of Management, Guangzhou University, Guangzhou Higher Education Mega Center, Guangzhou 510006, China; ^2^School of Mathematics and Information Science, Guangzhou University, Guangzhou Higher Education Mega Center, Guangzhou 510006, China

## Abstract

Since the outbreak of COVID-19, the rapid construction and operation of Wuhan Vulcan Mountain Hospital and Raytheon Hospital have attracted positive responses from local and international observers. At the same time, it has also highlighted the urgency for the construction of emergency medical facilities for public health emergencies. Before construction, the practical location of medical facilities is the basis for improving the city's emergency management ability. Based on the classic susceptible, exposed, infected, and recovered (SEIR) epidemic model and epidemic data in Guangzhou, we established a multi-stage time-delay SEIR epidemic model that is suitable for epidemic research in Guangzhou. According to the results of the model, the five areas with the highest number of infected patients were identified, which included Baiyun District, Panyu District, Haizhu District, Tianhe District, and Zengcheng District. We then centralized infected individuals at five demand points. Based on the distribution of these points and by combining the characteristics of the emergency medical facilities, we built and solved the set covering location decision model, and considered the economy, society, and environment as the starting points to optimize the site location. Finally, based on simulations, we concluded that appropriate site selection can increase the time required to reach the maximum number of patients and reduce the proportion of infected and exposed people by 11.3% and 1.11%, respectively. This is indicative of the effectiveness of the site selection model and the rational selection of facility points in this study. It solves the optimization problem of the location decision of emergency medical facilities for public health emergencies in China, and also provides some valuable references for site selection decisions of emergency medical facilities in other areas.

## 1. Introduction 

In recent years, SARS, avian flu, COVID-19, and other public health emergencies have occurred in relatively rapid succession, with an adverse effect of the loss of lives, safety, and property [1]. As society pays increasing attention to the safety of life and health, emergency rescue after a sudden disaster has become an issue of concern for society and the government. Starting from the end of December 2019, the novel coronavirus pneumonia epidemic has adversely impacted the global population [2–4]. In the case of critical care medical treatment, it is crucial to effectively control the development of the epidemic. China has taken unprecedented national interventions since January 23, 2020, including the establishment of emergency medical facilities to isolate and treat patients [5]. To accommodate patients and provide them with a better environment for treatment, the government decided to build the “Vulcan Mountain” and the “Raytheon Mountain” according to the hospital model of Xiao Tang Shan during the SARS period in 2003 [6]. The 1,000-bed Vulcan Mountain Hospital with a construction area of 34,000 m^2^ was completed in just 10 days and immediately began to treat its first batch of patients [5]. After Vulcan Mountain Hospital, the 1,600-bed Raytheon Hospital has been put into operation again, with a total of 10,000 discharged patients, making a great contribution in the fight against COVID-19 [7].

The establishment of emergency medical facilities, such as Vulcan and Raytheon Hospitals, for the segregation and treatment of patients, has played an indispensable role in curbing the progression of the epidemic. The success of the two hospitals indicates that the locations of emergency medical facilities must be considered including equity, adequacy, efficiency, accessibility, efficiency, overall population distribution, construction cost, coverage distance, and other influencing factors, in addition to scientifically based and reasonable decisions. As early as 2003, the Ministry of Health and the Ministry of Housing and Urban-Rural Development of China formulated the “Guidelines for the Architectural Design of Hospitals for treating SARS patients,” which stipulated that emergency medical facilities should be located in a flat area with high ground and stable geology, avoiding densely populated areas in accordance with the requirements of the overall urban plan. In February 2020, the National Health Commission with the Ministry of Housing and Urban-Rural Development of China created “COVID-19 contingency facilities design guideline,” which stated that a clear emergency medical facility location must meet the following condition: “the new location of the project should be located in good geological conditions, municipal facilities complete, convenient traffic location, and away from the densely populated areas. The site selection of the reconstruction and expansion project shall be located in the relatively independent area of the hospital with independent entrances and exits.” In December 2020, the Housing and Urban-Rural Development Department of Shandong Province and Health Commission of Shandong Province issued the “Guidelines for the Design of ‘Shelter Type' Temporary Emergency Medical Treatment Sites,” which stipulate that: (1) The site selection of temporary emergency medical treatment should be far away from high-density residential areas, kindergartens, large, primary, and secondary schools, and other urban crowded activity areas, as well as from inflammable, explosive, toxic, and harmful gas production and storage sites. The site selection should be downwind of the city's perennial dominant wind direction as far as possible. (2) There should be no less than 20 meters of separation between the existing building and the surrounding buildings. This shows that the Chinese government attaches great importance to the location of emergency medical facilities. The selection of an appropriate location can not only reduce costs but also ensure that in the event of a sudden disaster in the city, emergency medical service facilities can provide relief supplies and medical services to patients in the shortest time possible, thereby avoiding potential losses [8]. Therefore, to ensure a fraction of coverage, the determination of the optimal distribution of emergency service facilities in a specific area is of critical significance, and it is also a key link in emergency rescue services and the allocation of rescue resources.

The traditional set covering model is a type of deterministic model with a single factor. However, in real life, public health emergencies occur suddenly and with high uncertainty, so there is a random occurrence of accidents at each emergency demand point. In this study, we established a multi-stage time-delay susceptible, exposed, infected, and recovered (SEIR) epidemic model to analyze the existing epidemic data and forecast epidemiological trends, in combination with the actual situation and characteristics of emergency events by considering the spread of the virus, state intervention, and other factors that influence the location. The optimal solution could be obtained from the results of the analysis combined with the set covering model. Finally, the emergency medical facility location was determined scientifically. Moreover, the simulation results show that the site selection can better meet the urgent requirements of effective management of public health emergencies and also solve the problem of optimizing the layout of new medical facilities in the current urban planning and construction process.

## 2. Literature Review

In terms of the research on the virus propagation model, Tang et al. [9] established the stochastic discrete transmission dynamic model of COVID-19 using the limited data in the early stage, which provided an early reference for epidemic prevention and control [9]. Huang et al. [10] have provided a large amount of data for their research on COVID-19 epidemic control strategies [10]. Wang et al. [11] considered the SEIR epidemic model with time delay and impulse vaccination, and analyzed the global attraction of the disease-free periodic solution of the model [11]. Yan et al. [12] proposed a novel dynamic system with a time delay to describe the outbreak of NCP, the incubation period, and the treatment cycle, which was based on the cumulative number of diagnosed and cured cases reported daily by the National Health Committee. Numerical simulations revealed that the parameters in the model were accurately identified [12]. Din et al. [13] studied the mathematical model and transmission mechanism of COVID-19 and used a new type of epidemic model composed of four compartments: SEIR to describe the dynamics of COVID-19 under a convex incidence rate [13]. Zhang et al. [14] comprehensively considered the transmission characteristics of COVID-19, the impact of prevention and control interventions, medical conditions, the impact of medical resource availability, and other factors. They also considered the development process of the epidemic in China, extracted the correlation between prevention and control measures and the trend of the epidemic through parameter inversion, and established an empirical transmission dynamics method of COVID-19 analysis [14]. Medrek and Pastuszak [15] applied an enhanced infectious disease dynamics SEIR model to estimate the epidemic trend in Poland, France, and Spain, and the results showed the impact of the structure and behavior of the populations studied on key epidemic parameters [15]. Moreover, the obtained simulation results were in general agreement with the observed behavior of the COVID-19 disease, and the numerical framework was shown to effectively analyze the dynamics and efficacy of the epidemic containment method. Yang et al. [16] integrated the migration of data and the latest COVID-19 epidemiological data to the SEIR model, and used SARS data in 2003 to train artificial intelligence, which effectively predicted the peak and scale of the outbreak of COVID-19, and established its importance in reducing the COVID-19 outbreak in the implementation of control measures on January 23, 2020 [16].

In the study of emergency facility site selection, to reasonably select locations of emergency facilities, Wei et al. [17] proposed a site selection model that aimed to minimize the number of emergency facilities under the presupposition of covering all demand points [17]. Chen and You [18] considered the equity, efficiency, rapid response, and excess coverage of emergency rescue facilities, and proposed a multi-objective decision model that integrated the most used classical models for different emergency rescue facility deployment strategies [18]. Yang et al. [19] proposed an optimal location selection model for EMS facilities with multiple coverages and combined the model results with geographic information system analysis to show that the optimization of EMS location reduced the emergency response delay caused by disasters and significantly increased the coverage of rescue personnel and demand points [19]. First aid demands were combined with traffic states based on the spatiotemporal big data set covering model, which reduced the negative impacts of randomly occurring first aid demands and traffic delay time on the planning of pre-hospital first aid stations. In addition, this met the requirement that all randomly occurring simulated first aid demands can be approached within the target time under planning conditions and actual traffic constraints. The results of this study are expected to provide insight into the improvement of the site selection model and enhance overall efficiency, in addition to proposing new technical methods [20].

In terms of the location of emergency medical facilities, Hochbaum and Pathria [21] argued that the problem of siting emergency facilities must satisfy the maximum operating distance minimization over all time periods [21]. Mandell [22] considered a two-level EMS (Emergency Medical Service) system coverage model, which consists of two types of facilities with different processing capabilities [22]. Chu and Chu [23] proposed a framework for modeling the location of hospitals in Hong Kong and their resource allocation, which includes two aspects of optimization decisions [23]: (1) the selection of new hospital addresses and the redistribution of old hospital addresses and (2) the allocation planning of resources, including the setting of beds in the new hospital and the redistribution of beds in the old hospital. Their research results were successfully applied by the Hospital Authority in Hong Kong. The set covering model is commonly used in distribution center location in logistics for many-times operations; it is also suitable for emergency medical facilities location. Based on GIS, Deng et al. [24] used the nearest-neighbor approach to calculate the shortest travel time, applied location set coverage model to optimize the location of emergency medical facilities, and finally determined the optimal location by genetic algorithm [24].

Scholars from all over the world have conducted a lot of research on the viral infectivity model and the site selection of emergency facilities, but few of them have associated the site selection of emergency medical facilities with the viral infectivity model [25–30]. In this study, the SEIR model is innovatively combined with the improved set covering model to systematically study the location of emergency medical facilities and obtain the optimal solution.

## 3. Research Methods and Data Acquisition

### 3.1. Research Methods and Technical Route

**Figure 1:**
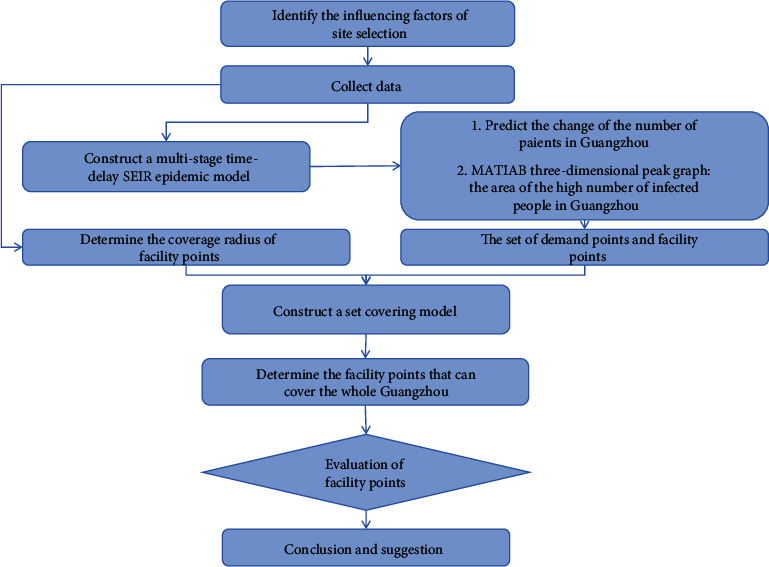
Technology roadmap.

As shown in Figure 1, considering the traditional emergency medical facility location model that lacks specific characteristics for the background of the epidemic outbreak of disease, the adopted approach combined quantitative and qualitative analyses, and established the SEIR epidemic dynamics model to simulate its state and law of development, and changes over time. In this report, we examine the set covering model that is based on the SEIR model, which provides a decision-making basis for emergency medical facility location selection, analysis methods, and methods of deployment. In addition, we use the model to effectively simulate disposal processes and allocate relief resources, reducing the threat of infectious disease outbreaks to life, health, and property.

The research procedure can be summarized as follows:

Step 1: Identify the factors that affect site selection prior to the study and collect a large amount of data.
Step 2: Construct a multi-stage time-delay SEIR epidemic model.
Step 3: According to the predicted results of the SEIR model, construct a three-dimensional peak graph of the peak area of the number of infected people in Guangzhou using MATLAB, and then determine the set of demand points and candidate facility points.
Step 4: The average radius of the original hospital coverage in Guangzhou is combined with the set of demand points and candidate facility points obtained in the previous step to establish the set covering model.
Step 5: MATLAB is used to solve the set coverage model, and the optimal solution is obtained according to the judgment matrix to determine the emergency medical facilities that can cover the entire region of Guangzhou.
Step 6: Combined with the actual conditions such as economic, social, and environmental constraints of the facility selection, a comprehensive evaluation method is adopted to evaluate, obtain the order, and determine the best facility points.
Step 7: Analyze and discuss the model results and draw conclusions and suggestions.

### 3.2. Data Acquisition

The acquisition of professional data is critical for facility site selection. The effectiveness and practicability of the site selection model are also based on the accuracy and completeness of the corresponding spatial and attribute data. The higher the level of detail and accuracy of the data, the more helpful it will be for spatial location selection. In this study, when solving the location problem, the data acquisition methods are as follows:

#### 3.2.1. Obtained from the Map

Obtaining relevant data from maps is the most intuitive approach. ArcGIS software contains various forms of relevant thematic maps, such as the distribution map of population resources, regional topographic maps, and regional meteorological monitoring maps. In addition, the Baidu map provides the traffic distance between demand points and facility points in this study using route planning, and its pickup coordinate system also helps to determine the longitude and latitude coordinates of each point.

#### 3.2.2. Text and Relevant Statistical Data

Each government department performs statistical analysis on the areas under its management and stores relevant statistics. For example, the epidemic data used in the establishment of the SEIR model in this study are from the epidemic notification column on the official website of the Guangzhou Municipal Health Commission [31]. The population size and distribution data were obtained from the official website of the Guangzhou Bureau of Statistics [32].

## 4. SEIR-Based Construction of Emergency Medical Facility Site Selection Optimization Model

### 4.1. Multi-Stage Time-Delay SEIR Epidemic Model

#### 4.1.1. Backdrop Characterization

SEIR is a classical mathematical model used to describe and predict the epidemic spread of infectious diseases. The main idea is to divide the population into four groups, namely, susceptible people *S*, exposed people *E*, infected people *I*, and recovered people *R*, and to establish ordinary differential equations through the infection mechanism of one group to another, to determine the law of virus transmission. The COVID-19 virus has a specific incubation period, and patients require hospitalization and observation from diagnosis to recovery. However, the classical epidemic dynamics model cannot reflect the time-delay relationship among patients with latent infection, diagnosed infection, and recovered patients, nor can it account for the influence of policy intervention measures and medical resources on the epidemic situation. It is easy for the forecast data to differ from the actual data, which leads to inaccuracies in the prediction of the development trend, the time to reach the peak, and the time required to eliminate COVID-19 in China.

Based on the comprehensive consideration of epidemic characteristics, intervention effects, and medical conditions, referring to the existing research and reference stage of discrete time-delay dynamics model [33–35], combined with the epidemic development process of Guangzhou, the classical infectious disease dynamics model was improved. Moreover, different models and parameters were used to express different stages, and a time-delay SEIR epidemic model was established, which was suitable for Guangzhou.

According to the daily epidemic data released by the Guangzhou Municipal Health Commission (Table 1), the newly diagnosed cases from January 21, 2020, to March 14, 2020, were all from Guangzhou. From March 14, 2020, to the present, almost all new cases have been imported from abroad. Due to the complexity and instability of overseas influencing factors, the epidemic data within this period are not included in the SEIR prediction model.

Therefore, the multi-stage time-delay SEIR epidemic model divides the development of COVID-19 in Guangzhou on March 14, 2020, into the following three stages:

(i) In the early stage of the outbreak (2020.01.20–2020.02.17), the rate of increase of infected persons gradually accelerated, and first-level responses to major public health emergencies were evident. The Guangzhou Eighth People's Hospital undertakes the prevention and control of major infectious diseases in the province, and the designated hospital has sufficient medical treatment and protection resources.
(ii) For the intense intervention control period (2020.02.18–2020.02.29), the growth rate of the number of infected people was stabilized, the number of newly infected individuals per day decreased significantly after the infection rate peaked, and disease control began to show results, but prevention and control measures were still strict, and medical resources were gradually depleted.
(iii) The emergency defense period (2020.03.01–2020.03.14), the number of new infections per day was reduced by an order of magnitude, and the epidemic situation was substantially stabilized. The level of prevention and control response was reduced, and some individuals were allowed to mobilize, whereas others insisted on isolation and protection.
(1) *Model Assumptions*.
  - The total population of Guangzhou is unchanged in 2020, and the birth rate and natural mortality rate will not be considered during the epidemic period.
  - Both the exposed and diagnosed infected individuals can infect susceptible persons.
  - In the isolation phase, everyone is quarantined without considering that recovered individuals can be reinfected.
  - All exposed patients have access to hospital care for treatment and become diagnosed infected people after the onset of symptoms, and can be hospitalized immediately after the diagnosis.
  - Exposed people can heal themselves.
  - If the body temperature of a diagnosed infected person returns to normal within 3 days after discharge and the retest is negative, he/she will be discharged from the hospital.  Table 2 describes the parameters of SEIR model and their instructions.

#### 4.1.2. Model Establishment and Solution

1. *Model Establishment*. A multi-stage time-delay SEIR model for Guangzhou was established by considering the epidemic characteristics, the impact of intervention, medical conditions, and the development process of the epidemic in Guangzhou. The natural population growth rate, mortality rate, and the number of deaths due to disease were not considered during the epidemic period in Guangzhou; thus, the transmission coefficient of diagnosed infected people was set as *B*, the number of patients who were contacted by susceptible people was set as *r*, the probability of exposed people was set as *a*, the recovery probability of diagnosed infected people was set as *y*, and *N* is the total population of Guangzhou.

Accordingly, the equations for the changes in the number of susceptible people S, exposed people *E*, infected people *I*, and recovered people *R* from time *t* to time *t* + 1 are shown in equations (1)–(4), respectively.

(1) $$\begin{array}{c} S\left(t+1\right)=S\left(t\right)-\frac{r\cdot B\cdot S\left(t\right)\cdot I\left(t\right)}{N}, \end{array}$$

(2) $$\begin{array}{c} E\left(t+1\right)=E\left(t\right)+\frac{r\cdot B\cdot S\left(t\right)\cdot I\left(t\right)}{N}-a\cdot E\left(t\right), \end{array}$$

(3) $$\begin{array}{c} I\left(t+1\right)=I\left(t\right)+a\cdot E\left(t\right)-y\cdot I\left(t\right), \end{array}$$

(4) $$\begin{array}{c} R\left(t+1\right)=R\left(t\right)+y\cdot I\left(t\right). \end{array}$$

Equations (1)–(4) can be solved using MATLAB by substituting the corresponding parameters. These equations correspond to the mathematical differential equations (5)–(8):

(5) $$\begin{array}{c} \frac{dS}{dt}=-\frac{rBSI}{N}, \end{array}$$

(6) $$\begin{array}{c} \frac{dE}{dt}=\frac{rBSI}{N}-aE, \end{array}$$

(7) $$\begin{array}{c} \frac{dI}{dt}=aE-yI, \end{array}$$

(8) $$\begin{array}{c} \frac{dR}{dt}=yI. \end{array}$$

The preceding are the equations corresponding to the four groups in the three stages.

The first stage (2020.01.20–2020.02.17): In the early stage of the outbreak, the rate of infection increased gradually, and the first-level response to major public health emergencies was enacted. The Guangzhou Eighth People's Hospital was tasked with the prevention and control of major infectious diseases in the province. This hospital had sufficient medical and protective resources. In this stage, the number of individuals who came into contact with diagnosed infected people was set as *r*_1_, and the recovery rate of infected individuals was set as *y*_1_. Accordingly, the equations for the changes in the number of susceptible people *S*, exposed people *E*, infected people *I*, and recovered people *R* from time *t* to time *t* + 1 are shown in equations (9)–(12), respectively.

(9) $$\begin{array}{c} S\left(t+1\right)=S\left(t\right)-\frac{r_{1}\cdot B\cdot S\left(t\right)\cdot I\left(t\right)}{N}, \end{array}$$

(10) $$\begin{array}{c} E\left(t+1\right)=E\left(t\right)+\frac{r_{1}\cdot B\cdot S\left(t\right)\cdot I\left(t\right)}{N}-a\cdot E\left(t\right), \end{array}$$

(11) $$\begin{array}{c} I\left(t+1\right)=I\left(t\right)+a\cdot E\left(t\right)-y_{1}\cdot I\left(t\right), \end{array}$$

(12) $$\begin{array}{c} R\left(t+1\right)=R\left(t\right)+y_{1}\cdot I\left(t\right). \end{array}$$

The second stage (2020.02.18–2020.02.29): Intense intervention and control period: the growth rate of the number of infected people was controlled, the daily number of newly infected people after the infection rate peaked was significantly reduced. Disease control was effective, prevention and control measures were still strict, and medical resources were gradually depleted. In this stage, the number of individuals who came into contact with diagnosed infected people was set as *r*_2_, and the recovery rate of infected people was set as *y*_2_. Accordingly, the equations for the changes in the number of susceptible people S, exposed people *E*, infected people *I*, and recovered people *R* from time *t* to time *t* + 1 are shown in equations (13)–(16), respectively:

(13) $$\begin{array}{c} S\left(t+1\right)=S\left(t\right)-\frac{r_{2}\cdot B\cdot S\left(t\right)\cdot I\left(t\right)}{N}, \end{array}$$

(14) $$\begin{array}{c} E\left(t+1\right)=E\left(t\right)+\frac{r_{2}\cdot B\cdot S\left(t\right)\cdot I\left(t\right)}{N}-a\cdot E\left(t-t_{1}\right), \end{array}$$

(15) $$\begin{array}{c} I\left(t+1\right)=I\left(t\right)+a\cdot E\left(t\right)-y_{2}\cdot I\left(t\right), \end{array}$$

(16) $$\begin{array}{c} R\left(t+1\right)=R\left(t\right)+y_{2}\cdot I\left(t\right). \end{array}$$

The third stage (2020.03.01–2020.03.14): Emergency defense period: the number of new infections per day was reduced by an order of magnitude, the epidemic situation was stabilized, the level of prevention and control response was reduced, and the mobility of individuals was improved. However, some individuals still insisted on isolation and protection. In this stage, the number of individuals who came into contact with diagnosed infected people was set as *r*_3_, and the recovery rate of infected people was set as *y*_3_. Accordingly, the equations for the changes in the number of susceptible people S, exposed people *E*, infected people *I*, and recovered people *R* from time *t* to time *t* + 1 are shown in equations (17)–(20), respectively:

(17) $$\begin{array}{c} S\left(t+1\right)=S\left(t\right)\;-\frac{r_{3}\cdot B\cdot S\left(t\right)\cdot I\left(t\right)}{N}, \end{array}$$

(18) $$\begin{array}{c} E\left(t+1\right)=E\left(t\right)+\frac{r_{3}\cdot B\cdot S\left(t\right)\cdot I\left(t\right)}{N}-a\cdot E\left(t-t_{1}\right), \end{array}$$

(19) $$\begin{array}{c} I\left(t+1\right)=I\left(t\right)+a\cdot E\left(t\right)-y_{3}\cdot I\left(t\right), \end{array}$$

(20) $$\begin{array}{c} R\left(t+1\right)=R\left(t\right)+\;y_{3}\cdot I\left(t\right). \end{array}$$

**Figure 2:**
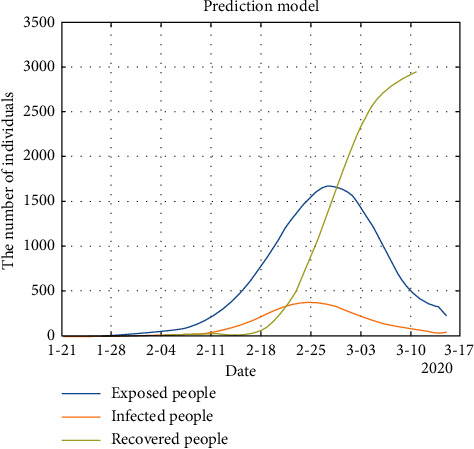
Prediction model.

(2) *Model Solution*. According to the differential equation for the preceding mathematical model, considering all the influencing factors and a large amount of available data, parameter estimation was performed, and the multi-stage time-delay SEIR epidemic model for Guangzhou was solved by modifying the existing MATLAB code. The prediction results are shown in Figure 2.

In Figure 2, the model predicts that the number of exposed people (*E*), the number of diagnosed infected people (*I*), and the number of recovered people (*R*) will increase slowly and synchronously from the first COVID-19 case recorded in Guangzhou on January 21, 2020, up to 20 days afterward.

Prediction: On February 11, the growth rate of the curves for the three groups of individuals began to accelerate. During the outbreak period, the number of exposed and infected people increased exponentially. On February 18, the growth rate reached its maximum, and the curve of the number of recovered individuals began to grow rapidly. The number of infected people reached a peak at approximately February 25 in Guangzhou, after which the curves of the number of exposed people (*E*) and the number of diagnosed infected people (*I*) began to decline. The outbreak ended on March 17.

**Figure 3:**
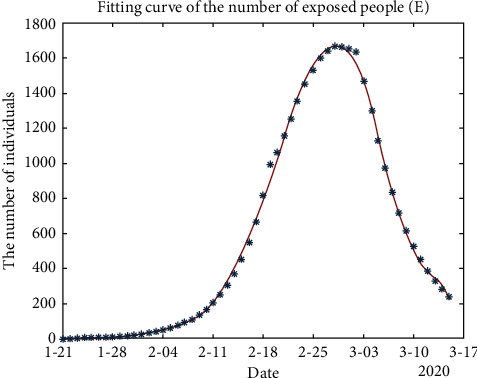
Fitting curve of the number of exposed people (*E*).

**Figure 4:**
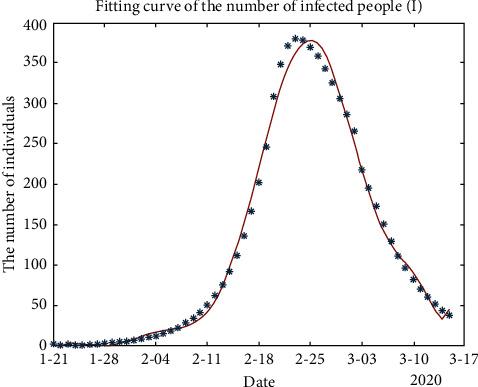
Fitting curve of the number of infected people (*I*).

In addition, in the process of solving the SEIR model using MATLAB, the fitting curve yielded the following three types of population curves of exposed, susceptible, and recovered individuals, respectively:

In Figures 3 and 4, the fitting curve of the number of exposed people (*E*) and infected people (*I*) are approximately normally distributed.  Figure 4 shows that the number of infected people increased to a maximum of 378.454 on February 25. From this result, we can predict that with the development of the epidemic, the important control period for Guangzhou is 55 days, and the outbreak will gradually stabilize after mid-March 2020. However, a period of strict control is still necessary to ensure success.

**Figure 5:**
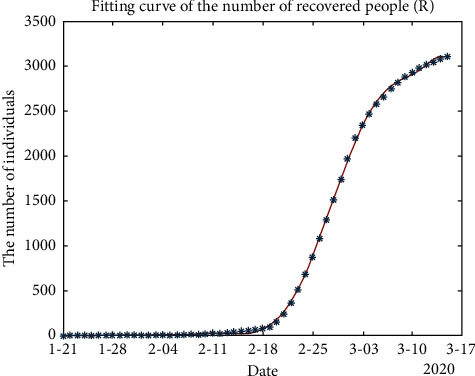
Fitting curve of the number of recovered people (*R*).

Figure 5 shows that February 18 was the turning point of the growth rate of the number of recovered people (*R*). Subsequently, the number of recovered people exploded exponentially, began to slow down on March 10, and the cumulative number of infected people remained stable.

#### 4.1.3. Model Validation

**Figure 6:**
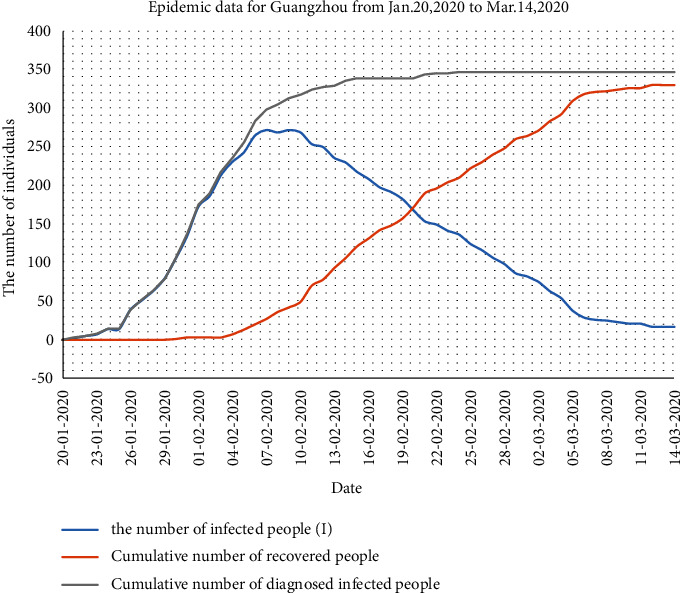
Epidemic data for Guangzhou from Jan. 20, 2020, to Mar. 14, 2020.

According to the daily epidemic notification data of the Guangzhou Health Committee, the epidemic data for Guangzhou from January 20 to March 14, 2020 (Table 6), were obtained, as shown in Figure 6. To ensure the correctness and rationality of the calculation results of the SEIR epidemic model, a comparative analysis of Figures 2 and 6 is performed in the following.

The epidemic data in Figure 6 shows that the curve of the number of infected people (*I*) is approximately normally distributed. The number of diagnosed infected individuals reached a peak value of 271 on February 7. In contrast to the result of the prediction model with actual data, we find that the curve of the number of diagnosed infected people (*I*) and the number of recovered people (*R*) deviate slightly from the curve of actual data. However, the overall trend is consistent with observation, which shows that the prediction model can accurately simulate the trend of the development of the epidemic, peak time, and end time in Guangzhou.

### 4.2. Location Optimization Model of Emergency Medical Facilities Based on SEIR

#### 4.2.1. Problem Description

The site selection of emergency medical facilities is different from that of the general public or private facilities that pursue the goal of maximizing profits. It must meet the requirements of fairness, sufficiency, high efficiency, accessibility, and economy, and realize the maximization of the distribution efficiency of medical and health resources and the fair distribution of resources among the population.

Based on the SEIR model, the maximum number of patients in each district of Guangzhou can be calculated, but the number of infected patients in most districts is negligible. As such, the number of infected individuals in a region with a large number of sick people was extracted and approximately converged on each demand point. The number of infected people at these demand points represents the number of people in the surrounding region. Based on the distribution of demand points and in considering the characteristics of fairness, sufficiency, efficiency, and accessibility of emergency medical facilities, a set covering model was established, and an algorithm was designed to solve the problem. Thus, the amount and location of emergency medical facilities were determined to achieve the maximum number of individuals in need using the minimum number of facilities. Moreover, we considered the economic, social, and environmental problems associated with the establishment of facilities, and introduced the cost influence factor, NIMBY factor, and pollution factor into the evaluation equation to evaluate and analyze the facilities. Finally, the effectiveness of the SEIR model and the rationality of facility site selection in this paper are verified through simulation, and the facility site selection scheme that can cover the whole infected area of Guangzhou as much as possible and meet various specific requirements is obtained (Table 3).

#### 4.2.2. Model Establishment and Solution

Step 1. *Identify the Demand Points.*

**Figure 7:**
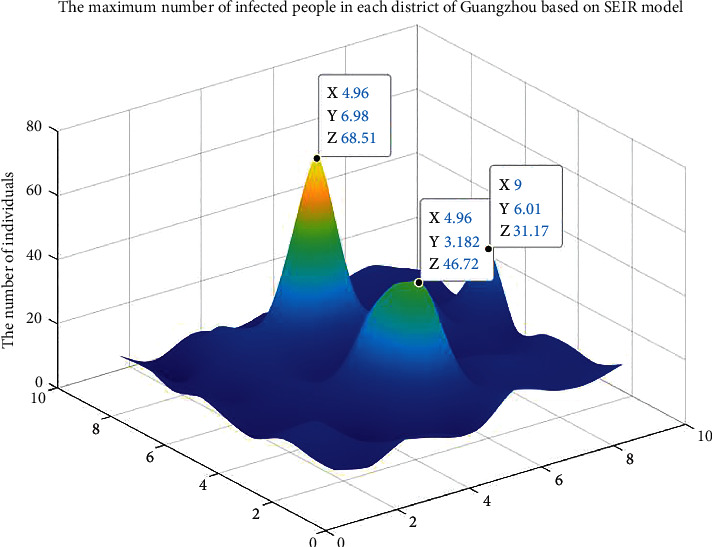
The maximum number of infected people in each district of Guangzhou based on the SEIR model.

As shown in Figure 7, based on the SEIR model, the three-dimensional peak figure of the number of infected people in each region of Guangzhou can be obtained. The *X* and Y values are converted to a certain proportion according to the geographical coordinates of each district of Guangzhou. For example, 4.96, 6.98 corresponds to Baiyun District and 4.96, 3.182 corresponds to Panyu District on the administrative district map of Guangzhou. The *Z* value is the maximum number of infected people in the corresponding region of Guangzhou predicted by the SEIR model. The specific method is as follows: based on the total population of the corresponding region, the maximum number of infected people in the region can be predicted by the SEIR model based on iteration in stages.

According to the three most significant peaks in Figure 7, the number of infected people reached a maximum at 68.51 in the Baiyun District, approximately 46.72 in the Panyu District, Haizhu District, and Tianhe District, and 31.17 in the Zengcheng District.

Therefore, the number of infected individuals in the five districts of Guangzhou converges on a demand point in each district. The coordinates of the points are as follows: Baiyun District (113.296, 23.221); Panyu District (113.390, 22.944); Haizhu District (113.324, 23.090); Tianhe District (113.377, 23.154); and Zengcheng District (113.817, 23.267).

**Figure 8:**
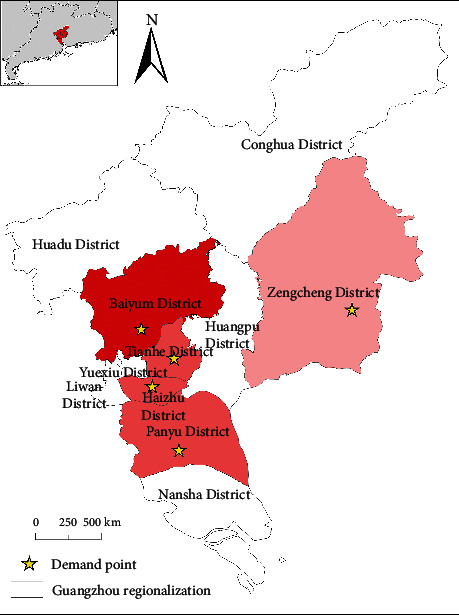
Map of demand points.

ArcGIS was used to mark the locations of the demand points on the map. As shown in Figure 8, the region with red color represents the peak value of the maximum number of infected people in the area, which means that the darker the color, the greater the number of patients.

Site selection of the facilities is a complex process. For public health emergencies, in addition to following the general principles of site selection, it also has the following characteristics:

1. Distance between emergency medical facilities and the city center with personnel activity concentration and heavy traffic.

Public health emergencies are extremely likely to cause panic due to their contingencies and infectiousness. When selecting facilities, city centers with a high concentration of personnel activity and heavy traffic should be avoided, and suburbs should be chosen that are as far away as possible.

(2) Distance from emergency medical facilities to roads with convenient traffic and stations, such as expressway exits and railway stops.

A convenient transportation network can depress influence and reduce the time required to rescue patients and transport supplies.

(3) Distance from emergency medical facilities to nature reserves.

Facilities should be located as far away as possible from natural areas and water sources assigned by the local government to reduce the contamination of natural resources by the virus.

(4) Emergency medical facilities should conform to land planning and be reasonably sized. They should not only meet the emergency needs of the current emergency but also take into account the need for reuse after the event.

Based on these principles of site selection for emergency medical facilities, we selected the following eight sites as candidates. Using optimization analysis of the set covering model, five superior facility points were selected from these candidate facility points: The west side of Yuexiu Park (113.264648, 23.142888); the east side of Dafushan Forest Park (113.344523, 22.954243); the east side of Pearl River Park (113.354845, 23.123592); the west side of Donghui City (113.805721, 23.289715); the northeast side of Laiyoulai fashion shopping mall (113.251154, 23.408798); the northeast side of Haizhu Gymnasium (113.287984, 23.086051); the northwest side of Guangzhou Horse Show Field (113.701487, 23.713701); and the southwest side of Baiyun Lake (113.243198, 23.232219). The distance between the demand point and the facility point was calculated, and the results are shown in Table 4.

Step 2. *Determine the Coverage Radius*. Taking the coverage of existing hospitals in Guangzhou as an example, the average radius of their coverage was calculated as the coverage radius of this model. The coverage radius is 3.6475.
Step 3. *Establish Set Covering Model*. The purpose of establishing emergency medical facilities is to meet the needs of all users, which means that we should consider the needs of the users in addition to both cost and utility. Therefore, we chose a set covering model to obtain the solution.
  - The set covering model determines a set of service facilities to meet the needs of the previously identified five demand points. In this model, the number and appropriate locations of service facilities must be confirmed to cover all demand points with a minimum number of facility points. In this step, a feasible solution was obtained by using a specific model according to a given area, and a set of solutions in closest agreement with the objective function was selected as the optimal solution of the model, which was used as the result of the problem of site selection.  Table 5 describes the parameters of set coverage model and their instructions, as shown in the following equations (21) and (22).

(21) $$\begin{array}{c} \text{Min}=\sum_{i\in C}X_{i}, \end{array}$$

(22) $$\begin{array}{c} \sum_{j\in N}x_{i}y_{ij}\geq 1,x_{i}=0\text{or}1,y_{ij}=0\text{or}1,i\in C,j\in N. \end{array}$$

Step 4. *Solve the Set Covering Model.*
  - The set covering model covers all the demand points with the minimum number of facility points, represents the demand points and facility points in the region with the set, and establishes the variable 0, 1 about *x*_*i*_, which means that 1 represents the establishment of the facility at point *i*, and 0 represents the opposite situation. *S*_*ij*_ represents the distance between facility point *i* and demand point *j*, and *L* represents the maximum coverage radius of the facility. As defined above with respect to the variable *y*_*ij*_, the judgment matrix was calculated using MATLAB, as shown in the following matrix formula (23).

(23) $$\begin{array}{c} \left|\begin{array}{cccccccc} 0 & 0 & 1 & 0 & 0 & 1 & 0 & 0\\ 0 & 0 & 1 & 0 & 0 & 0 & 0 & 0\\ 0 & 0 & 0 & 0 & 0 & 0 & 0 & 1\\ 0 & 1 & 0 & 0 & 0 & 0 & 0 & 0\\ 0 & 0 & 0 & 1 & 0 & 0 & 0 & 0\\ 0 & 0 & 0 & 0 & 0 & 0 & 0 & 0\\ 0 & 0 & 1 & 0 & 0 & 0 & 0 & 0\\ 0 & 0 & 0 & 0 & 0 & 0 & 0 & 0\\ 0 & 0 & 0 & 0 & 0 & 0 & 0 & 0\\ 0 & 0 & 0 & 1 & 0 & 0 & 0 & 0 \end{array}\right|. \end{array}$$

Therefore, the establishment of emergency medical facilities on the east side of Dafushan Forest Park (113.344523, 22.954243), the eastern side of Pearl River Park (113.354845, 23.123592), the west side of Donghui City (113.805721, 23.289715), the northeast side of Haizhu Gymnasium (113.287984, 23.086051), and the southwest side of Baiyun Lake (113.243198, 23.232219) can meet the medical needs of Guangzhou.

#### 4.2.3. The Evaluation of Facility Points

It is a complicated task to solve the problem of site selection, and many factors need to be considered. In terms of the economy, the establishment of hospitals is mainly related to coverage. The higher the demand for coverage, the higher the construction cost and maintenance protection cost. In terms of society, due to the suddenness and infectivity of public health emergencies, people are very likely to panic, and residents around the facilities may be sensitive to and even strongly resistant to emergency medical facilities, resulting in NIMBY risk. In terms of the environment, emergency medical facilities may have negative externalities. If medical waste is improperly disposed of, it may cause pollution and adverse effects on the ecological environment and residents around the city. Therefore, considering the economic, social, and environmental requirements, we established an evaluation equation to score each facility point to determine the optimal facility site selection scheme. Table 6 describes the parameters and instructions of the evaluation equation.

(24) $$\begin{array}{c} \omega =\alpha \frac{r_{k}}{r_{j}}+\beta \frac{d_{\text{max}}}{d_{z}}+\gamma \frac{p_{n}}{p_{i}}. \end{array}$$

As shown in Table 7, the evaluation scores of the five facility points calculated using MATLAB are 272.2923759, 285.5277479, 428.42417, 144.3277555, and 407.4453174. Thus, the optimum facility point is located on the northeast side of Haizhu Gymnasium, and the second one is located on the east side of Dafushan Forest Park, which both meet the economic, social, and environmental requirements of emergency medical site selection.

**Figure 9:**
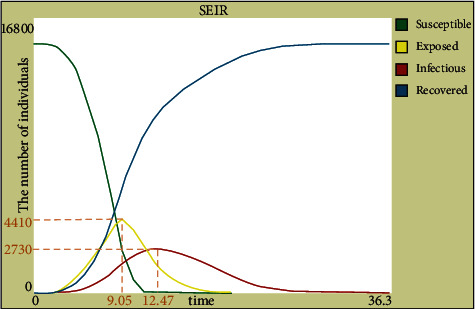
SEIR curve in the absence of emergency medical facilities.

**Figure 10:**
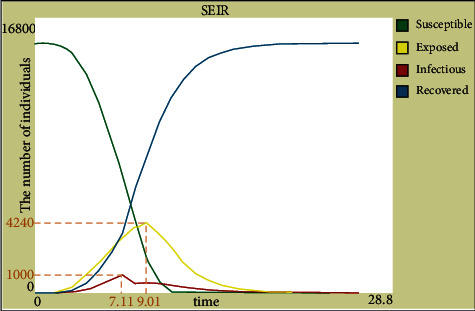
SEIR curve for setting up emergency medical facilities.

We then set the population in the NetLogo simulation program for 15300 people. The number of infected people and the number of exposed people were compared for the two cases to determine whether to establish emergency medical facilities. Without intervention, as shown in Figure 9, the proportion of exposed people reached 28.82% on day 9, and the proportion of infected people reached 17.84% on day 12. In the case of existing emergency medical facilities, as shown in Figure 10, the proportion of infected and exposed people peaked at 6.54% and 27.71% on days 7 and 9, respectively. Therefore, the results of this site selection can increase the time required for the number of patients to achieve a peak value and greatly reduce the number of infected and exposed people, further verifying the effectiveness of the results.

#### 4.2.4. Discussion

1. In terms of economy, the cost factors considered in the selection of candidate facilities include scale, quantity, transportation, and other factors. However, such medical facilities are mainly for public health emergencies, they need to be built in a short period. If the traditional patterns of project investment and financing costs are considered, this will delay the construction progress of facilities and have a negative effect. Second, the construction of emergency medical facilities is mainly subsidized by the state, and the construction cost has little influence on the site selection. Therefore, it is not a key consideration in the construction process of the site selection model. However, under correct guidance and supervision, we should actively encourage the construction of emergency medical facilities to adopt the PPP (public-private partnership) mode, and attract social capital to participate, alleviate the financial pressure of the government, realize the complementary advantages of the government and enterprises, and give full play to the effect of “one plus one is more than two.”
2. In terms of the environment, medical waste produced by COVID-19 is different from domestic waste, which is a type of hazardous waste that is highly infectious and attached to the external environment, and its remains may have deleterious effects. Influenced by the wind direction, topography, hydrology, and other factors, medical waste may cause pollution to the surrounding environment, such as water, atmosphere, and soil. Therefore, environmental factors were also considered in the process of site selection and included the following: First, stay away from natural protected areas and water sources to reduce the pollution of the natural environment and resources caused by the spread of the virus. Second, the manager of emergency medical facilities should formulate targeted emergency plans for each step in the process of collection, transportation, storage, and treatment of medical waste. After treatment, disinfection and air purification should be performed for medical waste containers or temporary storage rooms to avoid the occurrence of pollution accidents.
3. In terms of society, emergency medical facilities are also called NIMBY facilities. In the process of alleviating the pressure of treatment in designated hospitals and improving efficiency in fighting the epidemic, it may also have a series of negative external influences on the residents who live in the vicinity of the facility, and even cause additional problems such as environmental degradation, depression of local house prices, noise pollution, and other issues. A NIMBY incident can not only directly lead to the suspension of facility construction but will also cause an increase in social risk, ripple effects, and other secondary effects. Considering this point, we selected medical facilities with a low NIMBY risk that were far away from residential areas. Second, the construction of emergency medical facilities can also play an exemplary role for other provinces and even other countries. With existing hospitals operating at full capacity and new patients on the rise, the establishment of an independent infectious disease control hospital is the only logical option to ensure the safety of patients and doctors, and a specialized medical treatment system has proven to be of great help in improving the efficiency of treatment. The structure of a hospital cannot be designed overnight. Since the outbreak of SARS in 2003, China has acquired more experience than the United States and other countries in dealing with large-scale infectious diseases. Moreover, China's builders are willing to innovate, embrace new technologies, and use new construction methods, which also helped to improve construction speed. These experiences have been used in the construction of various emergency medical facilities during the spread of COVID-19 in China, providing western countries with an example of how to fight against the epidemic and promote social progress in emergency medical treatment.

## 5. Conclusion and Policy Recommendation

### 5.1. Summary of Results

After a major epidemic occurs, the establishment of emergency medical facilities and the early implementation of personnel protection, isolation, and treatment are key links and important means to meet the needs of emergency management, such as the rapid disposal, joint prevention, and control of national security effect incidents. COVID-19 is an infectious disease with a long incubation period and will have a notable effect in regions with a high degree of agglomeration, such as large cities and city clusters in China. Moreover, it represents a significant test of the national public health governance system and governance capacity, and triggers the need for emergency isolation and treatment space. Under such circumstances, the rapid construction of Wuhan Vulcan Mountain Hospital and Raytheon Hospital has been instrumental to COVID-19 prevention and control, demonstrating the importance of establishing a set of rapid, subregional, standardized, and intelligent emergency medical facility systems to alleviate the treatment pressure of designated hospitals and control the development of the epidemic. The location of emergency medical facilities is based on the distribution of the demand points. Considering the economy of the location of emergency medical facilities, we proposed that this could be realized by minimizing the number of emergency medical facility points. Given the problem of efficiency, we propose that this could be realized by maximizing the scale of emergency demand points covered by medical facility points.

We analyzed the target, principle, and influencing factors of the location of the emergency medical facility and established a multi-stage SEIR epidemic model, which assisted in the estimation of the largest number of infected people in each district of Guangzhou. The areas with a large number of infected people were centralized on each demand point, and the distribution of the demand points was used as the basis for the site selection of the set covering model to establish and solve this model. Based on the model results, the following conclusions can be drawn:

1. According to the multi-stage time-delay SEIR model, the coordinates of the demand points are calculated and used as the parameter basis of the set covering location decision model. The epidemic was under control for 55 days, and the inflection point appeared on February 25, when the number of infected people reached 378.454. The epidemic gradually became stable after mid-March 2020, which is consistent with the actual observation. The SEIR model can be used in MATLAB to draw the three-dimensional peak figure of the largest number of patients in each district of Guangzhou. It revealed that the five districts with the highest number of patients were Baiyun District, Panyu District, Haizhu District, Tianhe District, and Zengcheng District. The infected individuals in these five regions were approximately centralized at five demand points. The coordinates of the points were used as the parameter basis of the set covering model for site selection, and the two models were connected accordingly.
2. According to the set covering model, the number and appropriate location of emergency medical facilities are determined. First, the set covering model was solved using the MATLAB judgment matrix, and the quantity and appropriate location of medical facilities were determined in combination with the characteristics of fairness, adequacy, efficiency, and accessibility of emergency medical facilities. As such, the establishment of emergency medical facilities on the east side of Dafushan Forest Park, the eastern side of Pearl River Park, the west side of Donghui City, the northeast side of Haizhu Gymnasium, and the southwest side of Baiyun Lake can cover the medical needs of Guangzhou. Second, consideration of the economic, social, and environmental problems related to the construction of emergency medical facilities, we introduce the cost factors, NIMBY factors, and pollution factors into equation (24). Moreover, the comprehensive evaluation and analysis of such facilities are conducted. The evaluation scores of the five facility points (the east side of Dafushan Forest Park, the east side of Pearl River Park, the west side of Donghui City, the northeast side of Haizhu Gymnasium, and the southwest side of Baiyun Lake) were 272.29, 285.53, 428.42, 144.33, and 407.45, respectively. Among these, the one located on the northeast side of Haizhu Gymnasium is the best. The comprehensive evaluation results show that the constructed model and the solving algorithm of the model are feasible. Moreover, the solution results of the model can perfectly take into account the economic, social, and environmental properties of the site selection of urban emergency service facilities to facilitate the construction of urban emergency medical facilities with auxiliary site selection support.

### 5.2. Related Suggestions

Judging from the past, China has not only learned painful lessons but also accumulated valuable experience in dealing with public health emergencies. After the SARS epidemic, under the scrutiny of society, the design of the system of emergency medical facilities has been perfected via continuous exploration. In addition, the response and disposal capacity have improved, but there is still room for improvements, so we put forward the following suggestions:

1. Once an emergency occurs, personnel protection, isolation, and treatment should be implemented as soon as possible, and national prevention and control intervention measures should be strengthened so that the epidemic can be controlled rapidly.
2. Based on the current situation, the creation of a robust plan, taking a long-term view, careful preparation, assigning emergency medical facilities, and reserving the land for traffic control, municipal infrastructure, and other supporting facilities, is the basis for improving emergency prevention and control, epidemic prevention, and disaster relief.
3. Focus on the timeliness and accuracy of the release of COVID-19-related information, so that the public and society are well-informed, actively cooperate with the epidemic prevention and control work, and effectively participate in quarantine prevention and control measures. On the one hand, it is beneficial to the guidance of public opinion and policy interpretation; this promotes positive public sentiment and social stability. If the information is inaccurate, this may ignite negative social emotion, and increase the complexity of event triggers. Not only would this cause tremendous unpredictable risks and negative effects, it would even affect superior departments of the decision-making and guidance process, and can also cause the location decisions used in this report to produce deviation, resulting in a loss of applicability.
4. Develop a COVID-19 data processing method and prediction system based on machine learning to improve the SEIR model, so as to further improve the location optimization model of emergency medical facilities. The results can be used to provide reference for decision-making and reduce the loss of people's life and property safety.
5. In terms of the environmental friendliness of site selection, the possible negative externalities of emergency medical facilities should be avoided and positioned as far as possible on the edge of the city. To reduce the adverse impacts on the ecological environment and the residents of the city, it is necessary to establish the downstream downwind direction, and adequate safety exclusion zones around the city should be identified to meet the requirements of environmental assessment and safety assessment.

## Figures and Tables

**Table 1:** COVID-19 epidemic situation in Guangzhou (refer to daily epidemic data of Guangzhou Municipal Health Commission).

Date	New number of diagnosed infected people	Cumulative number of diagnosed infected people	The number of infected people (*I*)	New number of recovered people	Cumulative number of recovered people
2020/1/20	0	0	0	0	0
2020/1/21	2	2	2	0	0
2020/1/22	3	5	5	0	0
2020/1/23	2	7	7	0	0
2020/1/24	7	14	14	0	0
2020/1/25	0	14	14	0	0
2020/1/26	25	39	39	0	0
2020/1/27	12	51	51	0	0
2020/1/28	12	63	63	0	0
2020/1/29	16	79	79	0	0
2020/1/30	27	106	105	1	1
2020/1/31	31	137	134	2	3
2020/2/1	38	175	172	0	3
2020/2/2	14	189	186	0	3
2020/2/3	27	216	213	0	3
2020/2/4	21	237	230	4	7
2020/2/5	18	255	242	6	13
2020/2/6	29	284	264	7	20
2020/2/7	14	298	271	7	27
2020/2/8	6	304	268	9	36
2020/2/9	9	313	271	6	42
2020/2/10	4	317	268	7	49
2020/2/11	6	323	253	21	70
2020/2/12	4	327	249	8	78
2020/2/13	1	328	235	15	93
2020/2/14	7	335	229	13	106
2020/2/15	3	338	217	15	121
2020/2/16	1	339	208	10	131
2020/2/17	0	339	197	11	142
2020/2/18	0	339	191	6	148
2020/2/19	0	339	182	9	157
2020/2/20	0	339	167	15	172
2020/2/21	4	343	153	18	190
2020/2/22	2	345	149	6	196
2020/2/23	0	345	141	8	204
2020/2/24	1	346	136	6	210
2020/2/25	0	346	124	12	222
2020/2/26	0	346	116	8	230
2020/2/27	0	346	106	10	240
2020/2/28	0	346	98	8	248
2020/2/29	0	346	86	12	260
2020/3/1	0	346	82	4	264
2020/3/2	0	346	75	7	271
2020/3/3	0	346	63	12	283
2020/3/4	0	346	54	9	292
2020/3/5	1	347	38	17	309
2020/3/6	0	347	29	9	318
2020/3/7	0	347	26	3	321
2020/3/8	0	347	25	1	322
2020/3/9	0	347	23	2	324
2020/3/10	0	347	21	2	326
2020/3/11	0	347	21	0	326
2020/3/12	0	347	17	4	330
2020/3/13	0	347	17	0	330
2020/3/14	0	347	17	0	330

**Table 2:** Parameters of SEIR model and their instructions.

Parameter	Instruction
*S* (*t*)	The number of susceptible people at time *t*
*E* (*t*)	The number of exposed people at time *t*
*I* (*t*)	The number of diagnosed infected people at time *t*
*R* (*t*)	The cumulative number of recovered people at time *t*
*t*_1_	The time delay of incubation period
*t*_2_	The time delay of treatment observation period
*A*	The probability of exposed people
*B*	The transmission coefficient of diagnosed infected people
*r*	The number of patients who are contacted by susceptible people
*r*_1_	The number of patients who are contacted in the first stage
*r*_2_	The number of patients who are contacted in the second stage
*r*_3_	The number of patients who are contacted in the third stage
*y*	The recovery probability of diagnosed infected people
*y*_1_	Stage 1, the recovery rate of infected people when medical supplies are scarce
*y*_2_	Stage 2, the recovery rate of infected people when the medical supplies are fully prepared
*y*_3_	Stage 3, the recovery rate of infected people when the medical supplies are sufficient

**Table 3:** Relevant data of 11 administrative regions of Guangzhou.

Serial number	Municipal districts	Area/km^2^	Permanent resident population/10,000 people	Population density/(10,000 people km^2^)
1	Yuexiu district	33.80	120.97	3.58
2	Liwan district	59.10	101.20	1.71
3	Haizhu district	90.40	172.42	1.91
4	Tianhe district	96.33	178.85	1.86
5	Baiyun district	795.79	277.96	0.35
6	Huangpu district	484.17	115.12	0.24
7	Panyu district	786.15	182.78	0.23
8	Huadu district	970.04	110.72	0.11
9	Nansha district	527.65	79.61	0.15
10	Zengcheng district	1616	126.01	0.08
11	Conghua district	1974.50	64.95	0.03
	Total number	7433.93	1530.59	

**Table 4:** Distance between demand point and facility point (unit: km).

	The west side of Yuexiu Park	The east side of Dafushan Forest Park	The east side of Pearl River Park	The west side of Donghui City	The northeast side of Laiyoulai fashion shopping mall	The northeast side of Haizhu Gymnasium	The northwest side of Guangzhou Horse Show Field	The southwest side of Baiyun Lake
Haizhu district	12	23	7.5	71	44	6.5	93	25
Tianhe district	15	28	6.4	65	41	19	83	23
Baiyun district	12	39	18	68	29	20	86	9.4
Panyu district	35	6.5	24	76	66	25	108	50
Zengcheng district	69	72	66	4.7	75	70	66	76

**Table 5:** Set coverage model parameters and their descriptions.

Parameter	Instruction
*N*	The set of demand points in the region, *N* = {1,2,…, *n*}
*C*	The set of candidate facility points that can be established in the region, *C* = {1,2,…, *c*}
*x*_*i*_	0–1 variable. If the facility point is established at point *i*, *x*_*i*_=1, otherwise, *x*_*i*_=0
*S*_*ij*_	Distance from facility point *i* to demand point j
*L*	Maximum coverage radius of the facility
*y*_*ij*_	0–1 variable, if *S*_*ij*_ ≤ *L*, *y*_*ij*_=1, otherwise, *y*_*ij*_=0

**Table 6:** The parameters and instructions of the evaluation equation.

Parameter	Instruction
*ω*	Evaluation score of facility point (The smaller the *ω*, the better the evaluation of the facility point)
*α*	Cost influence factor, *α* = {1,2,…, 10}
*r*_*k*_	Average distance between facility point and residential area
*r*_*j*_	Coverage radius of the facility point
*β*	NIMBY factor, *β* = {1,2,…, 10}
*d*_max_	Maximum distance from the facility point to a nearby residential area
*d*_*z*_	Average distance from all facility points to nearby residential areas
*γ*	Pollution factor, *γ* = {1,2,…, 10}
*p*_*n*_	Covers the area of residential areas
*p*_*i*_	Total pollution of covered area

**Table 7:** *ω* Value calculation table.

Facility point	The east side of Dafushan Forest Park	The east side of Pearl River Park	The west side of Donghui City	The northeast side of Haizhu Gymnasium	The southwest side of Baiyun Lake
*ω*	272.2923759	285.5277479	428.42417	144.3277555	407.4453174

## Data Availability

The datasets used and/or analyzed during the current study are available from the corresponding author on reasonable request.
